# Whether two heads are better than one is the wrong question (though sometimes they are)

**DOI:** 10.1007/s10459-020-09956-z

**Published:** 2020-02-06

**Authors:** Wolf E. Hautz, Stefanie C. Hautz, Juliane E. Kämmer

**Affiliations:** 1 Department of Emergency Medicine, Inselspital University Hospital, University of Bern, Freiburgstrasse 16c, 3010 Bern, Switzerland; 2 Institute of Health and Nursing Science, Charité – Universitätsmedizin Berlin Corporate Member of Freie Universität Berlin, Humboldt-Universität zu Berlin, Berlin Institute of Health, Oudenarder Str. 16, 13347 Berlin, Germany; 3 Center for Adaptive Rationality (ARC), Max Planck Institute for Human Development, Lentzeallee 94, 14195 Berlin, Germany

A recent editorial by Norman (2019) in this journal asked whether “[t]wo heads are better than one”. Following a light-hearted and insightful deliberation on medical training specifically and problem solving in general, either individually or in groups, Norman concluded that “two (independent) heads are better than one (group of two heads)” (2019). We applaud the author for questioning a widely accepted belief and for fostering a discussion on the pearls and pitfalls of collaboration in medicine. Drawing on a review of the medical and psychological literature, we would, however, argue that his conclusion (a) leaves important evidence out of consideration, (b) results from a conceptual oversimplification, and (c) addresses the wrong question. In the following, we highlight relevant research on the merits of one versus more heads in the context of medical diagnoses, present a theoretical conception of the problem, and conclude that the question of whether or not to collaborate should be replaced by that of when and why to collaborate, aggregate or work in isolation. We conclude with specific suggestions for further research in this area, illustrating our point with an example from research into group leadership.

## What is the evidence?

As Norman (2019) notes, ample research on collective intelligence or the wisdom of the crowd shows that two (or more) independent heads are better than one: Algorithmically aggregating two or more opinions usually outperforms the average (e.g., Kämmer et al. 2017; Kurvers et al. 2015; Surowiecki 2005) and sometimes even the best *individual* (Wolf et al. 2015). The paper by Barnett et al. (2019), which sparked Norman’s editorial and which he skillfully dissects, reports similar findings, albeit challenged by methodological limitations and an unusual use of the term “group” (Norman 2019).

Given the relevance and ubiquity of teamwork today (Deloitte Insights 2019), the more pressing question is perhaps indeed—as Norman (2019, p. 197) suggests—how two *independent* heads compare with two *interacting* ones. We addressed precisely this question in a recent experimental study (Hautz et al. 2015), in which advanced medical students individually or in interacting teams of two diagnosed virtual patients presenting to the emergency room. Teams were more accurate than individuals (67.78 vs. 50.00%; difference 17.78% [95% CI, 5.83–29.73%]; *P* = .004), although knowledge levels were comparable and equal numbers of diagnostic tests were consulted before a diagnosis was made (Hautz et al. 2015). Most importantly for the current discussion, we found that interacting teams outperformed the same number of independently working individuals whose solutions were algorithmically aggregated. Specifically, we randomly paired students who had participated individually into simulated pairs (or “nominal groups”) and used the diagnosis of the more confident member as this pair’s diagnosis. We repeated this procedure 1000 times. The mean accuracy of simulated pairs (mean 56.73%; 95% CI, 49.72–63.74%) was comparable with that of individuals (mean 50%; 95% CI, 40.53–59.47%) but below that of real pairs (mean 67.78%; 95% CI, 59.95–75.6%; *F*(2,83) = 6.75, *η*_*p*_^2^ = 0.12; *P* = .002) (Hautz et al. 2015). Further simulations (Kämmer et al. 2017) showed that it would have taken aggregation of the opinions of three independent individuals according to a follow-the-most-confident rule [i.e., maximum confidence slating (Koriat 2012)] to outperform interacting pairs. Admittedly, this approach relies on the assumption that confidence is related to diagnostic accuracy on a per-case basis. In an analysis of a heterogeneous sample of 283 students working through the same cases individually, students were, on average, indeed more confident in diagnoses that were correct (mean confidence 57.3%; 95% CI, 54.2–60.3%) than in those that were not [41.8%, 95% CI, 39.1–44.6%; *F*(1,253) = 196; *P* < .001; *d* = .63)] (Hautz et al. 2019)—a result that is in line with other findings on self-monitoring (Eva and Regehr 2011; Pusic et al. 2015; Tweed et al. 2017).

In sum, evidence for the merits of aggregating independent heads (Kämmer et al. 2017; Kurvers et al. 2015; Surowiecki 2005) represents just part of the picture. A number of studies (Hautz et al. 2015; McMahon et al. 2016; Navajas et al. 2018) also provide evidence for interaction being beneficial to performance. Admittedly, these studies are still few in number, and more research is required into when and why interaction can outperform the wisdom of crowds.

## How to conceptualize the problem?

In his editorial, Norman (2019) first discusses individual versus group-based *learning*, explicitly contrasting lectures to small group instruction. Although lectures are typically held in front of very large groups, they are often seen as individual instructions. This somewhat paradoxical classification results from the theoretical explanation, that the benefits of collaborative learning largely result from interaction with socially and cognitively congruent peers (Lockspeiser et al. 2008). Lectures are simply assumed to be too large to allow for such peer interaction. Norman later extends his argument to *performance* tasks such as diagnosis. However, the type of task heavily affects the answer (c.f., Soderstrom and Bjork 2015): learning may occur in solitude (e.g., through contemplation, observation, or reading) or in groups of various sizes. In learning groups, others merely constitute the environment that enables (or hinders) learning, which ultimately occurs in the individual. For learning tasks, it is thus possible to meaningfully compare individuals who studied and perform alone with those who studied in groups but then perform alone. Several studies of motor skill learning conducted such comparisons and report —at least for complex skills—non-inferiority in effectiveness (and thus higher efficiency) of small-group versus individual learning (e.g., Räder et al. 2014; Tolsgaard et al. 2015).

In performance tasks, in contrast, the group is both the environment *and* the performing entity. With the possible exception of blatant individual errors, it is next to impossible to attribute the outcome of an interacting team’s performance to any individual member of that team. Consequently, the performance of groups cannot be meaningfully compared with that of individuals in isolation in performance tasks [but we are guilty of reporting such comparisons ourselves (Hautz et al. 2015)]. Even if we do compare the performance of nominal groups of size *n* to that of interacting groups of the same size (rather than to the average or best individual), it remains an abstract comparison that offers only limited insights. Indeed, such a comparison might amount to comparing “treatment of myocardial infarction with aspirin and nitroglycerin to treatment with low-molecular weight heparin, primary angioplasty, beta-blockade, angiotensin-converting enzyme inhibitor, HMG-CoA-reductase inhibitor, clopidogrel, and folate—all at once. Even if a significant result is found in such an investigation, little is known about which therapies contributed” (Cook 2005, p. 542, referring to cross-media comparisons). Likewise, even if differences are found in the diagnostic performance of groups and individuals (whether alone or aggregated into nominal groups), it is impossible to say whether they are due to the number of people involved or result from specifics of the groups’ configuration, behavior or the mode of aggregation. While nominal and interactive groups might both take advantage of an increasing amount of resources (such as knowledge, skills, cognitive capacity or experience) with increasing group size, interactive groups are affected by a number of phenomena that remain absent in nominal groups. On the downside, interactive groups have to coordinate their members, a demand that may result in process and motivation losses (Steiner 1972). In addition, mechanisms such as group conformity bias [the urge of a group member to conform with the stance of a majority (Kiesler and Kiesler 1969)], groupthink [group harmony taking priority over decision quality (Baron 2005; Janis 2013)], and polarization [groups taking more extreme decisions than any of their individual members is initially inclined to (Moscovici and Zavalloni 1969)] may reduce group performance even further. On the other hand, discussions among group members may yield new emergent solutions (“more than the sum of its parts”) because of the combination of non-redundant information, for example (Kerr and Tindale 2004). Thus, even if the size and composition of nominal and interactive groups is kept equal, a comparison between the two—despite providing a necessary benchmark in many instances—can yield only vague insights into the origins of performance differences and is thus of limited use.

## Why is it the wrong question?

Finally, we argue that the question of whether or not to work with colleagues on a particular problem is irrelevant in clinical practice. There, doctors either work in the seclusion of a private practice or are part of a clinical team (from the tumor board to ward rounds to multidisciplinary trauma teams). Although research may inform how such teams are formed and run, in most cases it would be difficult to substitute them by minds working in isolation (with or without later aggregation into a nominal group). The ever-changing demands of the health care environment will further increase the need for collaboration (Committee on Diagnostic Error in Health Care et al. 2015). Driving factors include increased specialization, with no single individual having all knowledge relevant to a given patient’s case, the accelerating rate of technological change, growing economic pressures resulting in the deregulation of health care, which necessitates collaboration across professions, and older patients with more complex conditions requiring multidisciplinary attention. All of these factors make individual practice increasingly difficult or even obsolete.

The rather black-and-white question of whether or not to collaborate to solve tough problems should nowadays be answered with a clear “it depends.” As Norman (2019) notes, the idea of using brainstorming in interactive groups to solve virtually every problem (Osborn 1953) was later abandoned entirely in favor of individual decision making (Stroebe and Diehl 1994). But throwing out the baby with the bathwater in this manner was not the route to optimal performance either. The current recommendation is to combine individual and group phases of decision making, depending on the problem (Paulus and Kennworth 2019; Stroebe and Diehl 1994; Van De and Delbecq 1971). A combination or iterations of individual and group phases may likewise be suited for numerous healthcare problems. The diagnostic process, for example, comprises phases of information gathering, interpretation, and integration (Committee on Diagnostic Error in Health Care et al. 2015). Here, it may be advisable to consciously delegate specific phases to either groups or individuals. It remains an empirical question for future research which combination yields the best results for which task.

While most of this commentary is concerned with *performance* tasks, we suspect that our conclusion may also apply to individual versus group based *learning*. As Norman notes (2019), evidence for the effectiveness if small group instruction is mixed. Studies that focus on *knowledge* acquisition often reveal negative effects (Davis et al. 1992; Eagle et al. 1992). However, some studies that focus on the acquisition of *procedural skills* report rather positive consequences of small group instruction (e.g., Räder et al. 2014; Tolsgaard et al. 2015). Again, whether or not groups should be preferred over individuals seems to depend on the specifics of the task (such as learning verbal material or performing a motor task; see e.g. Soderstrom and Bjork 2015).

## What are the consequences?

In a thorough discussion of the epistemological basis of medical education, Norman (2003) suggested that “the various factors that might contribute to a result [be] systematically varied over a series of experiments, based on a theory of causation, so that the real active ingredients in the treatment are understood” (p. 584). Decades of research, predominantly from sociology and social psychology, have provided plenty of theories on group performance that could and should be tested in their application to clinical environments. For example, a recent review concluded that the four factors information distribution, information exchange, heterogeneity in experience, and information retrieval from the team by its members all crucially affect performance in collaborative clinical reasoning (Kiesewetter et al. 2017). The role of these and other factors, however, is likely context depended. As just one example, consider team leadership. Teams diagnosing an unstable patient’s condition in a trauma room benefit from directive leadership behavior, and “talking to the room” is associated with longer time to a correct diagnosis (Härgestam et al. 2016). In the context of ambiguous presentations in non-life-threatening situations, in contrast, collaborative leadership and talking to the room are associated with better performance (Tschan et al. 2009). In a review of the literature on how to lead trauma teams, Ford et al. (2016) emphasized the context dependency of leadership and concluded that “directive leadership is most effective when Injury Severity Score (ISS) is high or teams are inexperienced, while empowering leadership is most effective when ISS is low or teams more experienced” (Ford et al. 2016).

A thorough, theory-guided and methodologically sound investigation of when, why and how which type of teams (or individuals, for that matter) are best applied to which clinical problems may provide more valuable insights (Hautz et al. 2017) than a discussion about the general superiority of individuals, nominal or interacting groups.
